# Acceptance of a Digital Assistant (Anne4Care) for Older Adult Immigrants Living With Dementia: Qualitative Descriptive Study

**DOI:** 10.2196/50219

**Published:** 2024-04-19

**Authors:** Marloes Bults, Catharina Margaretha van Leersum, Theodorus Johannes Josef Olthuis, Egbert Siebrand, Zohrah Malik, Lili Liu, Antonio Miguel-Cruz, Jan Seerp Jukema, Marjolein Elisabeth Maria den Ouden

**Affiliations:** 1 Technology, Health & Care Research Group Saxion University of Applied Sciences Enschede Netherlands; 2 Department of Technology, Policy, and Society Faculty of Behavioural, Management and Social Sciences University of Twente Enschede Netherlands; 3 Faculty of Humanities Open University Heerlen Netherlands; 4 Care & Technology Research Group Regional Community College of Twente Hengelo Netherlands; 5 Research Group Ethics and Technology Saxion University of Applied Sciences Enschede Netherlands; 6 School of Public Health Sciences Faculty of Health University of Waterloo Waterloo, ON Canada; 7 Department of Occupational Therapy Faculty of Rehabilitation Medicine University of Alberta Edmonton, AB Canada; 8 GRRIT Hub Glenrose Rehabilitation Research Innovation & Technology Glenrose Rehabilitation Hospital Edmonton, AB Canada; 9 Smart Health Research Group Saxion University of Applied Sciences Enschede Netherlands

**Keywords:** assistive technology, technology acceptance, immigrant, dementia, marginalized older adults

## Abstract

**Background:**

There is a need to develop and coordinate dementia care plans that use assistive technology for vulnerable groups such as immigrant populations. However, immigrant populations are seldom included in various stages of the development and implementation of assistive technology, which does not optimize technology acceptance.

**Objective:**

This study aims to gain an in-depth understanding of the acceptance of a digital personal assistant, called Anne4Care, by older adult immigrants living with dementia in their own homes.

**Methods:**

This study used a qualitative descriptive research design with naturalistic inquiry. A total of 13 older adults participated in this study. The participants were invited for 2 interviews. After an introduction of Anne4Care, the first interview examined the lives and needs of participants, their expectations, and previous experiences with assistive technology in daily life. Four months later, the second interview sought to understand facilitators and barriers, suggestions for modifications, and the role of health care professionals. Three semistructured interviews were conducted with health care professionals to examine the roles and challenges they experienced in the use and implementation of Anne4Care. Content analysis, using NVivo11, was performed on all transcripts.

**Results:**

All 13 participants had an immigration background. There were 10 male and 3 female participants, with ages ranging from 52 to 83 years. Participants were diagnosed with an early-stage form of dementia or acquired brain injury. None of the older adult participants knew or used digital assistive technology at the beginning. They obtained assistance from health care professionals and family caregivers who explained and set up the technology. Four themes were found to be critical aspects of the acceptance of the digital personal assistant Anne4Care: (1) use of Anne4Care, (2) positive aspects of Anne4Care, (3) challenges with Anne4Care, and (4) expectations. Assistance at first increased the burden on health care professionals and families. After the initial effort, most health care professionals and families experienced that Anne4Care reduced their tasks and stress. Contributions of Anne4Care included companionship, help with daily tasks, and opportunities to communicate in multiple languages. On the other hand, some participants expressed anxiety toward the use of Anne4Care. Furthermore, the platform required an internet connection at home and Anne4Care could not be used outside the home.

**Conclusions:**

Although older adult immigrants living with dementia had no previous experience with digital assistive technology specifically, the acceptance of the digital personal assistant, called Anne4Care, by older adult immigrants living with dementia was rather high. The digital assistant can be further developed to allow for interactive conversations and for use outside of one’s home. Participation of end users during various stages of the development, refinement, and implementation of health technology innovations is of utmost importance to maximize technology acceptance.

## Introduction

### Background

Dementia is a global health problem associated with emotional and financial challenges for people living with dementia, their relatives, health care professionals, and health organizations [1,2]. Worldwide, approximately 47 million people are diagnosed with dementia [3]. Around 280,000 persons are presently living with dementia in the Netherlands among a population of nearly 19 million [4]. The number of people living with dementia is expected to increase in the coming years. People with an immigration background are at an increased risk of developing dementia and are more likely to experience barriers in accessing dementia services and care, which may lead to health inequities and a reduction of quality of life [5-7]. Hence, there is a need to develop and coordinate dementia care plans, including the use of technology, for marginalized older adults living with dementia, such as those with an immigration background and low literacy levels [8].

### Health Literacy and Health Technology

Health literacy has been defined as “the degree to which individuals have the capacity to obtain, process, and understand basic health information and services needed to make appropriate health decisions” [9]. Adequate health literacy and access to services among immigrant populations are important to empower, support or facilitate these persons to become active participants in their health [10]. Persons with limited health literacy have difficulty finding, understanding, and applying information about health and health care. Health technology–based solutions that support health promotion, for example, mental and physical health, offer opportunities to increase health literacy in vulnerable populations [11,12].

The World Health Organization reports that innovative health technologies are promising tools to enhance knowledge, skills, and coping mechanisms to improve the daily lives of people with dementia and their caregivers [13]. In recent years, a variety of technologies to support self-management have been developed for people with dementia and their caregivers. Studies have focused on the effect of these technologies on the physical, mental, and social well-being of people with dementia and their relatives and families [14,15]. Technology seems to stimulate cognitive function and communication skills and reduce loneliness among people with dementia, but these results are personal and context-dependent [14,15]. Acceptance of technology is a major challenge and prerequisite for the implementation of technology in practice. For successful technology implementation in the daily lives of people, it is important to have insight into the acceptance of technology among end users. Although studies have examined the acceptance of technology among people living with dementia and their caregivers [16-18], the number of studies that focus specifically on older adult immigrants living with dementia is minimal.

### Citizen Science

Co-design and the involvement of people with dementia and their professional and family caregivers are crucial elements for the acceptance of technology [17]. However, immigrant populations are seldom included in stages of development, refinement, and implementation of assistive technology innovations [19]. This can be associated with low levels of acceptance of health technology innovations, which can contribute to health inequities among populations. One opportunity to increase the involvement of end users is through citizen science, which is an approach that engages end users to be partners in research so that their experiences and needs are considered [20]. Citizen science is a powerful approach to include public participation in research as well as optimize acceptance of technologies [20,21]. In citizen science, scientific principles and methods are used by nonprofessional “scientists” in close collaboration with scientific researchers [22]. The involvement of older adults in the development, refinement, and implementation of technology, acknowledging their expertise and needs, and working together in short iterations to adapt the technology for their specific needs are reported to be valuable elements by scientific researchers, older adults, and health care professionals [23].

### Aim

This qualitative descriptive study aimed to provide an in-depth understanding of the acceptance of a digital personal assistant, called Anne4Care, among older adult immigrants living with dementia by using a citizen science approach.

## Methods

### Research Design

This study used a qualitative descriptive research design with a naturalistic inquiry [24] and citizen science principles [20]. In this study, Anne4Care was extended with the development of a Turkish version. All the involved older adults had the task of testing the device as well as communicating with each other, their care professionals, and the Anne4Care help desk. For example, there were workshop-like meetings in which all shared experiences, assisted each other with difficulties, and expressed the need to further develop the Turkish version of Anne4Care. Furthermore, participants actively collaborated with scientific researchers, for example, in discussing the topics for the interview guide and analyzing the data from the interviews.

Aligning with citizen science principles there was a close collaboration between older adults participating as co-researchers, health care professionals, and scientific researchers. A detailed description of how older adults with an immigration background were engaged in this study is described in a separate paper [23]. The team of researchers closely collaborated with a group of citizens representing the target group and health care professionals in their role as co-researcher, during all phases of this study: recruitment, obtaining informed consent, data collection, and analysis.

### Setting

This research was part of the TOPFIT Citizenlab program, a research and innovation program in which citizens, health care professionals, and companies join forces with scientific researchers to develop and implement technology for health.

### The Technology

Anne4Care is a digital personal assistant that includes video-calling, a personal agenda, medication reminders, reading the news, and games that can be used in one’s home. Anne4Care is a technological platform created to help people with dementia to continue living independently in their own homes and supporting caregivers in their tasks [25]. Anne4Care included hardware as well as a software platform. Anne4Care is available in Dutch, German, Italian, and English. The company was developing a Turkish version of Anne4Care and saw the embedding of Anne4Care in homes of older adults with an immigration background as an opportunity to test, improve, and implement the latest version.

### Recruitment Strategy

The recruitment of participants was performed by 3 health care professionals from 2 health care organizations. These organizations provide care for clients with an immigration background with cognitive impairments.

Inclusion criteria were as follows:

- Diagnosed with an early-stage form of dementia or acquired brain injury;
- Having an immigrant background;
- Visiting the activity program of 1 of the 2 participating health care organizations

Exclusion criteria were as follows:

- Diagnosed with a severe stage form of dementia limiting their ability to participate

All clients of the 2 health care organizations (IMEAN Consultancy & Care and Alifa Wellbeing Older Adults) were invited by their health care professionals to participate in this study. All older adult participants had an immigration background, that is, 1 came from Britain and the other 12 came from Turkey (Table 1). They visited the activity program of 1 of the 2 health care organizations, which are situated in the Twente region of the Netherlands. In addition to an immigration background, all 13 older adult participants, or co-researchers, had an early-stage form of dementia or acquired brain injury. The sample included 10 male and 3 female participants, and their ages ranged from 52 to 83 years. There were no exclusion criteria based on digital literacy, that is, participants did not need any experience with technology. Internet access was provided to participants who did not have an internet connection at home.

**Table 1:** Demographic characteristics of the participants (N=13).

Variables		Values
**Age (years)**		
	Mean (SD)	71 (9.8)
	Range	52-83
**Age group (years), n (%)**		
	45-54	1 (7.7)
	55-64	1 (7.7)
	65-74	6 (46.2)
	75-84	5 (38.5)
**Sex at birth, n (%)**		
	Female	3 (23.1)
	Male	10 (76.9)
**Nationality, n (%)**		
	Turkish	12 (92.3)
	British	1 (7.7)

All 3 health care professionals involved in this study were female. They introduced Anne4Care to the older adults, assisted them, and were in close contact with the participants during all phases of this study. The health care professionals took care of the clients and spoke their native language, which created a safe and trustful environment. The health care professionals also served as a voice for the participants who could not communicate in the Dutch language.

### Data Collection

Anne4Care was introduced to allow health care professionals to communicate with and monitor older adults. Data were collected between September 2020 and November 2021. Semistructured in-depth interviews took place at the care organization or at the home of the older adult immigrants. The location was chosen based on a participant’s preference. During the interviews, a care professional was present and served as an interpreter. Participants were invited for 2 interviews. The first interview was planned shortly after the introduction of Anne4Care in their home, and a second interview 4 months later. Participants had the opportunity to continue using Anne4Care after the data collection period, funded through a stimulation subsidy for eHealth at home during the COVID-19 pandemic. Five researchers with mixed credentials, training, occupation, location, and gender conducted the interviews (MB, CMvL, TJJO, ES, and ZM). To secure interrater reliability, the researchers had biweekly meetings to discuss the procedure and previous interviews. Two researchers were present during each interview; the teams of 2 were different for each interview. One was the main interviewer and the other took notes and asked additional questions. The follow-up interviews were conducted by the same researchers to ensure the established relationship between researchers and older adults.

The topic guide was developed in collaboration with 1 participant from the daycare facility and 3 health care professionals. Thirteen first interviews were conducted focusing on understanding the lives and care needs of the participants, learning about their expectations regarding Anne4Care, and sharing previous experiences with care and technologies, what facilitators and barriers they encountered while using technology (Multimedia Appendix 1). The first interviews lasted between 30 and 60 minutes. A total of 8-second interviews were conducted with the same participants who participated in the first interview (5 of the participants were not able to participate in the second interview due to COVID-19 illness). The second interview focused on the facilitators and barriers participants experienced when using Anne4Care, suggestions for modifications of Anne4Care, and the role of health care professionals in using technology (Multimedia Appendix 1). The second interview lasted between 20 and 45 minutes.

Three semistructured interviews were conducted with the 3 health care professionals. These interviews took place at the care organization and lasted for 60 minutes. The aim of these interviews was to talk about their role and the challenges they experienced as professionals in the use and implementation of Anne4Care (Multimedia Appendix 2). Furthermore, the findings of the interviews with the older adults were shared with the care professionals.

### Data Analysis

All interviews were audio recorded and transcribed verbatim. The transcripts were made in English and Dutch. All Turkish spoken words were translated by an interpreter during the interviews. Only these translations were part of the transcripts. Content analysis of transcripts used an inductive approach [26,27]. We used the software package NVivo11 to support data coding. Open coding was used to identify relevant themes, there were no themes in advance of the data analysis. Several steps were taken to develop a code book. First, 3 researchers (MB, CMvL, and TJJO) performed the analysis of 2 transcripts and compared codes. A preliminary codebook was developed comprising these themes. Second, the coding of one transcript was discussed together with one older adult. This participant was motivated and had some previous experience with research and data analysis. Together with the co-researcher, additional themes were added to the code book. Last, the other transcripts were analyzed by 1 researcher (CMvL). The data analysis and application of codes were discussed during biweekly meetings with the research team. Data saturation was reached after analyzing the data obtained with all involved older adults and care professionals. During the data analysis, similar and confirmation of all findings appeared when analyzing and coding the transcripts. In preparation for the paper, the quotes in the raw data were translated into English.

### Ethical Considerations

Ethics approval was obtained from the ethical advice committee of the University of Applied Sciences Saxion (reference number SEAC-2020-005). The participants were informed about the study before the start of the research period with Anne4Care. Thirteen participants gave written consent and were informed about their right to withdraw at any time. Data were anonymized, confidentiality was maintained, and the data will be retained for a period of 10 years after which they will be destroyed.

## Results

### Overview

The analysis of data revealed that the personal situation and perceptions of participants regarding access to dementia services and care (in short: care) were 2 underlying themes that described the adopter system from the older adult immigrants living with dementia. The code tree is presented in Multimedia Appendix 3.

### Personal Situation

Personal situation considers the perceptions of older adult immigrants regarding their health status (physically and mentally), level of spirituality, quality of life, social and societal participation, and daily functioning. The personal lives of participants were diverse, with their immigration background as a main commonality. They were all born in another country and moved to the Netherlands for their work. Most of the participants worked in the textile or metal industry. The participants were all retired and visited the facility for daytime activities once or twice a week. On other days, their daily activities consisted of grocery shopping, housekeeping, or just doing nothing. Spirituality (eg, religion) was an important part of the lives of most participants. The physical and mental health of all participants is deteriorating, with illnesses that range in severity. In addition to dementia or acquired brain injury, some have health conditions such as diabetes, high blood pressure, and cataracts. These deteriorating health conditions had a major impact on their daily functioning as well as their quality of life. In the earlier years of their lives, some participants experienced challenges in participating in Dutch society, but this became even more difficult during the COVID-19 pandemic. Social relations were mainly with family members, and some had close contact with their neighbors.

Since I came here in the Netherlands, in the year 1977, I bought a house and since then until now I live in the same street, the same neighborhood with the same neighbors and this was always a very good network. It is a community with the Turkish and Dutch neighbors, in which I am a beloved man, I go to the mosque a lot and had several board positions for a while, such as a board member of the mosque.

Participant H

### Care

Care refers to the perceptions of participants regarding access to dementia services and care in including all assistive and care needs of the participants. All participants acknowledged the fact that they became older and had increasing trouble taking care of themselves. Some participants had troublesome experiences with care in the past or could not find suitable and personalized care. In most cases, family members, partners, children, or neighbors assisted and provided support and care. However, most participants perceived this as a burden for their relatives and, therefore, was not a desirable situation.

Now there is someone who assists in housekeeping, and our children assist with the more administrative tasks. Next-door there are some younger neighbors who offered to help for example with the garden. I have a lot of help from all of them. However, in the past I had a lot of frustrations with health care professionals. When we arranged a time, they did not show up and none made any record of my needs. Then the agency went bankrupt and the clients were left in the dark.

Participant G

### Acceptance of Anne4Care

In addition to the 2 underlying themes, the analysis of the acceptance of Anne4Care by older adult immigrants revealed four themes: (1) use of Anne4Care, (2) positive aspects of Anne4Care, (3) challenges with Anne4Care, and (4) expectations. In this section, results are presented for each theme. The code tree is presented in Multimedia Appendix 3. Multimedia Appendix 4 provides visual information about Anne4Care.

### Use of Anne4Care

This theme refers to the acceptance and actual use of the digital personal assistant Anne4Care by older adult immigrants living with dementia in their own homes. The use of Anne4Care applies to how someone uses Anne4Care, what someone does with Anne4Care, and how health care professionals or families are involved. None of the participants knew or used an assistive technology similar to Anne4Care. All were unfamiliar with the existence of these types of digital assistive technologies. One participant acknowledged that technology like Anne4Care could assist health care professionals. In addition, the health care professionals underlined the potential of Anne4Care for supporting clients at a distance. Although the participants were unfamiliar with technologies like Anne4Care, they were familiar with technologies such as a doorbell with a flashlight or a talking clock, and 1 participant owned a robot vacuum cleaner.

I was so pleased when I saw the result, this robot really cleans everything. You can just leave your home and it will clean everywhere.

Participant L

Anne4Care was mainly used as a memory assistive tool, for appointments and medication.

It is very useful. Anne4Care tells me when I must take my medication. She helps me to remembers, she is tough and fun. I am very happy with it.

Participant M

In addition to the agenda function and medication reminders, the game, radio, and newspaper functions of Anne4Care were used or requested by some participants. A health care professional would need to be aware of participants’ requests in order to activate the radio or newspaper functions.

He would like to receive more radio channels. He has a Turkish music channel, but would like to receive Dutch channels as well. We can add these channels easily to the list, I will ask him at a later moment which he would prefer.

Health care professional B

Thus, the health care professionals and sometimes family were responsible for adding new functions as well as appointments in the agenda, and changes in the medication list. In the beginning, this costed time and was a source of burden. Some assistance to understand Anne4Care was needed at the start. However, after this initial adjustment, most health care professionals and families experienced the tablet as a task relieving as well as stress relieving. Anne4Care gave the reminders so that care partners did not have to keep track of everything during the day. Some participants admitted that continuous reminders from their partners made them angry, but reminders from Anne4Care were received more positively, causing fewer troubling situations at home. Furthermore, some participants preferred to update the agenda themselves if they could learn to work with Anne4Care. This feature is currently not possible with the platform.

I just need a keyboard to add appointments in my agenda. It is important for me to do this myself without any assistance, just some explanation and exercises in the beginning. It would be great if that would work!

Participant E

After a few months, 3 participants decided to stop using Anne4Care. Two participants did not see the additional value, Anne4Care did not give them any new tools, and 1 participant stopped using them due to illness.

### Positive Aspects of Anne4Care

This theme refers to participant experiences regarding the advantages and benefits of Anne4Care. During the interviews, participants were asked to share the positive aspects of Anne4Care. The avatar of Anne4Care was received positively by the participants. Coincidentally, “Anne” is also the Turkish word for mother. Although Anne does not look like a Turkish mom, she gave a feeling that there was someone in their homes because she talks, makes movements, and looks like a nurturing health care professional. Another positive aspect was the choice of language. Most participants chose their native language because Dutch was progressively more difficult to use with age and since the onset of dementia. However, some participants chose the Dutch language intentionally in order to develop and maintain their Dutch language skills.

With regard to positive aspects, the participants commented on the functionalities of Anne4Care: video calling, agenda, medication reminders, games, radio, and newspaper. The most positive aspect of the video calling was the quality and the size of the screen (respectively 10-12.3 inch diagonal). They could see the other person more clearly on the tablet than, for example, on their smartphone. The most positive aspect about Anne4Care was the agenda function with the reminders of appointments and the medication reminder function. These reminders were very essential for the participants’ personal life and health. One health care professional explained that any event or task could be added.

Take for example the timing for their regular prayers. These are essential for someone’s life, and we can easily add these into the agenda.

Health care professional

Another participant talked about the assistance of Anne4Care in the daily cooking routine.

There are reminders when I need to start cooking, but also already before which groceries I have to purchase. After a while Anne4Care asks: ‘did you turn off the stove?’ That is very helpful and important for me.

Participant K

The game function was experienced as a fun activity to do during the day. Furthermore, the radio and newspapers provided by Anne4Care were perceived to be valuable. For example, some radio channels with music from their past gave the participants an opportunity to escape from their current time and place. The newspapers were seen as essential to keep up to date with current events; the read-aloud option was an asset.

It is wonderful that the radio and newspapers can provide the news into my home. The news keeps me up to date, and I also know what happens in Turkey where my family is.

Participant H

### Challenges With Anne4Care

After using Anne4Care, the participants were asked about any challenges they experienced. As mentioned, the agenda and medication reminder functions were experienced as positive. However, the video calling option presented challenges. The main challenge was in making a connection with others. A video call required both the caller and receiver to activate Anne4Care. Therefore, all participants had to first send an SMS text message with their mobile phone to request the recipient to activate Anne4Care in order to receive a video call. With this extra step, most participants decided to simply use their mobile device instead of Anne4Care to make a video call. In addition, the newspaper and radio functions do not allow participants to search for radio stations, other than the ones preprogrammed.

I cannot find that newspaper, also the radio channel is absent. When I try to search, I get the massage ‘no stations available’, so there is nothing programmed I think.

Participant B

Another challenge was real interactions with Anne4Care. The participants expected the possibility to have a conversation, but that was not possible. Furthermore, part of this interaction was the commands to which Anne4Care often does not react. For example, when a participant asked Anne4Care for the time, or to call someone, Anne4Care may not respond. This could have been caused by the fact that Anne4Care did not recognize all the verbal commands in the Turkish language.

We cannot talk together, because she does not respond. Every morning I hear ‘good morning’, but that is it.

Participant B

The Anne4Care device itself presented some challenges. One challenge was the anxiety among participants for the devices to overheat, which caused the participants to turn off Anne4Care. Another challenge was the requirement for an internet connection at home. Some of the participants did not have internet at the start of the study. Internet access was provided to participants who did not have an internet connection at home during the study period. However, internet access is not free. Some of the participants are strapped for cash. This makes it difficult for them to pay for Anne4Care and an internet connection when the study ends. They have to make difficult trade-offs.

Now we are using Anne4Care for free, but in a couple of weeks there are probably some costs involved. We do not have Internet connection. I only have a mobile phone subscription with which I am happy. But I am also happy with Anne4Care, so the costs make it quite difficult to make a trade-off.

Participant N

Because Anne4Care is now only available with an internet connection at home, another challenge is to receive messages from Anne4Care when someone is outside. It would be great, for example, to transfer this with the Anne4Care message app on their mobile phone in case they are not at home. According to some participants, Anne4Care is currently a device only for people who are at home most of the day.

### Expectations

This theme represents the ideas, wishes, and future plans of the participants for Anne4Care. In the beginning, some of the participants expressed anxiety toward Anne. They turned the tablet off at night because they thought someone could see or listen to them through the device. One of the new plans most of the participants came up with during the use of Anne4Care was the addition of an option to connect quickly to emergency care services. This connection could be activated by the user, but it should also be activated automatically when older adult immigrants living with dementia do not respond to a call within a period of time. Although all participants expected that Anne4Care would improve the health care of people with deteriorating health or dementia, their expectations were higher at the start.

Anne4Care needs to be improved. At the moment, it is too basic and does not meet the needs of some people. We can do more by ourselves, it is a bit of a disappointment.

Participant D

As mentioned, it is a challenge to use Anne4Care outside the home because the platform requires the internet. Outside the home, it would be helpful if people could receive medication reminders, therefore, this platform should also be compatible with their mobile devices.

It would be great if I could just take Anne4Care outside. Then I have my medication reminders when I am outside, she will tell me to take the medication and I could take them at the right moment.

Participant M

There were different ideas for new functions on the current Anne4Care tablet. For example, the addition of short movies or documentaries would allow it to be used for entertainment. Additions to the game function and more options, such as multiplayer games allow an older adult to play with a partner. A range of memory or language-related games, and more challenging puzzles would help meet user preferences. The memory and language games were specifically mentioned by several participants and health care professionals, because of the perceived benefits of cognitive engagement for older adult immigrants living with dementia.

## Discussion

### Summary of Findings

This study aimed to understand the acceptance of Anne4Care as perceived by older adult immigrants living with dementia using a citizen science approach. This study showed that although older adult immigrants living with dementia had no previous experiences with digital assistive technology specifically, the acceptance of the digital personal assistant, called Anne4Care, by these participants was rather high. Anne4Care was mainly used as a memory assistive tool, for appointments and medication. The use of Anne4Care at first increased the burden of health care professionals and families because health care professionals and sometimes families were responsible for adding new functions as well as appointments in the agenda and changes in the medication list. After the initial effort, most health care professionals and families experienced that Anne4Care reduced their tasks and stress. Contributions of Anne4Care included companionship, help with daily tasks, and opportunities to communicate in multiple languages. On the other hand, some participants expressed anxiety toward the use of Anne4Care and experienced challenges in the use of functionalities, for example, video calling and having real interaction with the avatar. Furthermore, the platform required an internet connection at home and Anne4Care could not be used outside the home.

### Reflection on the Literature

The personal digital assistant, Anne4Care, offered companionship for older adult immigrants living with dementia and helped them perform daily activities. Participants mentioned that Anne4Care was very useful as a remember assistive tool, especially for appointments and medications. This finding is consistent with the results of previous studies about technology acceptance in rehabilitation and assistive technologies, and in health care technologies in general [28-30]. Technology acceptance models, for example, the Unified Theory of Acceptance and Use of Technology suggest that if performance expectancy is high, that is, people believe that technologies can help them to achieve their therapeutic goals or achieve their health expectations, this increases the acceptability and actual use of health technologies [31].

A key finding in this study was that participants believed that they did not have all the conditions to use and adopt Anne4Care for a longer period. These conditions are also known as facilitating conditions in technology acceptance theories, for example, the Unified Theory of Acceptance and Use of Technology [31]. Facilitating conditions include, for example, internet connection, technical infrastructure, as well as other internal support such as health care professional involvement and supporting staff (eg, availability of engineers to support the system) [32,33]. In our study, participants reported a lack of internet connection as a main limitation for the acceptance of Anne4Care. There is an extensive body of literature in the field of technology acceptance and use that points toward facilitating conditions as an important determinant factor in technology use [34]. Our result shows that to take full advantage of the potential of digital technologies like Anne4Care, these digital technologies should be accessible also to people of lower socioeconomic status which may have an influence on a person’s autonomy and independence [35].

In our study, health care professionals were involved in using Anne4Care. These health care professionals had close contact and a trustful and respectful relationship with the participants. They played an important role in giving participants information and instruction regarding Anne4Care, in which they can be supportive of the acceptance of technology. Some participants were concerned about their privacy when using Anne4Care. They turned the tablet off at night because they thought someone could see or listen to them through the device. The important role of the health care professionals in this study warrants discussion. The health care professionals, all with a Turkish background, had the expertise to provide access and involve the participants. They were an important frame of reference and guided the participants whenever they received questions, or observed discomfort or doubts. Furthermore, they were key users of health care technology and had a positive attitude toward implementing the new technology. Research suggests that creating a positive, supportive atmosphere is instrumental to the sustainability of technology use [36]. Other studies describe that caregiver engagement is important for the everyday use of technology among people with dementia [33,37].

Although in recent years several technologies have been developed for people with dementia and their caregivers to support self-management, in our study none of the participants knew or used an assistive technology comparable to Anne4Care. All were unfamiliar with the existence of these types of technologies. Globally, there is an increasing aging population and more people staying longer in their own homes which has an impact on society and health care [38]. Although evidence shows that technology for people with dementia seems to stimulate cognitive function and communication skills and reduce loneliness [14,15,39], the implementation of technology can also contribute to the burden of health care professionals and informal caregivers. In our study, participants needed the assistance of health care professionals or families, which increased the demand for health care professionals and families. However, after this initial effort, most health care professionals and families experienced that the tablet facilitated their caregiving roles.

Immigrant populations typically have limited involvement in the development, refinement, and implementation of health care technology. This may be reflected in lower levels of adoption of health care technology. The citizens’ science approach within this study was important for this specific target group. A citizen science approach calls for the optimal involvement of the target group as co-researchers. This extra time and dedication was positively experienced by the older adult immigrant group. It was mentioned that it felt like having a real purpose and gave a sense of fulfillment. This collaboration resulted in an alignment of the technology with the needs and practices of the participants. In future studies, the citizen science approach could also be applied in the development of new technologies as this study focused on the refinement of a technology for a specific target population. Citizen science for health and well-being could provide an effective way to involve vulnerable groups within society to participate in research.

### Recommendations for Future Research

Future research could examine ways to increase the implementation of technology among older adult immigrants living with dementia and how to develop the technology competencies of clients, caregivers, and health care professionals. Furthermore, for future research conducting mixed method research using both qualitative and quantitative research methods is recommended to provide more insight into the added value of these kinds of assistive technologies for end users and professionals or family members.

### Strengths and Limitations

Although we included only 13 older adults, they were involved in the entire process and collaborated with the researchers as well as with the other participants and their health care professionals. All older adults of the 2 organizations with whom the company Anne4Care started collaboration in the Twente region were invited for this study. The 13 participants who were interested in collaborating were all involved in this study. Another strength of our study was the quadruple collaboration. In our citizen science approach, there was active collaboration between the researchers, older adults, health care professionals, and the company that developed Anne4Care. However, we only involved older adults with an early-stage form of dementia, and 5 older adults were not able to join the second interview, due to COVID-19 illness. In addition, assessing exact levels of health literacy would have provided additional information about the influence of these kinds of assistive technologies on health literacy levels. Finally, a care professional was present and served as an interpreter. This may have impacted the quality of the data collected and nuances in the conversations may have been missed. On the other hand, the presence of the care professional ensured a safe and trustworthy environment.

In this study, a qualitative descriptive research design with a naturalistic inquiry has been used. Four themes were found to be related to acceptance of Anne4Care: (1) use of Anne4Care, (2) positive aspects of Anne4Care, (3) challenges with Anne4Care, and (4) expectations. Data saturation regarding the acceptance of Anne4Care was reached. During the data analysis, similar and confirmation of all findings appeared when analyzing and coding the transcripts. However, besides these 4 themes, there might be other factors relevant to the acceptance of digital personal assistant technology for older persons to stay safe in their homes and be able to age in place.

### Conclusions

Although older adult immigrants living with dementia had no experience with digital assistive technology specifically, the acceptance of the digital personal assistant, called Anne4Care, by older adult immigrants living with dementia was rather high. In our study, older adult immigrants living with dementia learned and used Anne4Care with the help of family caregivers. Most older adults accepted Anne4Care into their lives in which Anne4Care offered companionship and helped them to perform daily activities. Older adults provided suggestions for the continued development of Anne4Care.

## Appendix

Multimedia Appendix 1 Interview guide for first and second interviews with older adults with an immigration background.

Multimedia Appendix 2 Interview guide for interviews with health care professionals.

Multimedia Appendix 3 Coding tree.

Multimedia Appendix 4 Visual information about Anne4Care.
