# Supplementary material for: Acceptance of a Digital Assistant (Anne4Care) for Older Adult Immigrants Living With Dementia: Qualitative Descriptive Study
Source: JMIR Aging. 2024 Apr 19;7:e50219. doi: 10.2196/50219 (PMC11069095; doi:10.2196/50219)
Supplement: Multimedia Appendix 1 [file aging_v7i1e50219_app1.docx]

**Appendix 1. Interview guide & follow-up interview guide – older adults with an immigration background**

This is the English translation of the interview guide that was used both during the first and follow-up interviews. The introductory questions were only used during the first interview. The follow-up questions regarding technology and the question regarding collaboration and participation in the project were only asked during the follow-up interviews. The questions about Anne4Care and questions for informal caregivers were used during the first as well as the follow-up interviews.

*Introduction*

- Can you tell us something about yourself?
  - Age
  - Educational level
  - Nationality
  - Living situation
  - Social network
  - Health status
  - Informal caregivers
- What do you usually do in a day?
  - How does your day look like?
  - What do you like to do in your life?
- What is important to you regarding your personal health?
  - What is your definition of health?
  - How would you rate your own health (on a scale of 1-10)?
  - What could contribute to improving your health; what do you need?
- Do you use technology or digital tools in daily life?
  - What technology (digital tools) do you use at home?
  - And in your life with dementia (think of mobile phones, fall prevention, alarm bell, video bell application etc.)?

*Questions about Anne4Care.*

- When did you receive Anne4Care?
- How do you experience Anne4Care?
  - Pleasant / unpleasant?
  - Helpful / unhelpful?
- How have you used Anne4Care so far?
  - Can you give examples of your use of Anne4Care?
  - Can you show the functions you have used and how?
- Which advantages do you experience with Anne4Care?
- Which disadvantages do you experience with Anne4Care?
- What are improvements for Anne4Care?
- Would you like to continue using Anne4Care?
  - What are your reasons to continue / discontinue using Anne4Care?
- Do you need Anne4Care in your life?
  - Could you elaborate on that?
- What is the added value of Anne4Care (daily structure, maintaining social contacts, performing meaningful day activities)?
- Do you need (or have had) help in using or understanding Anne4Care?
  - If so, from whom?
  - Do you need more support or training in the] use of Anne4Care?
    - If so, from whom?
- Do you expect your health or your daily life to change because of using Anne4Care?
- Do you think Anne4Care will change your relationship/contact with your health care professional?
- Do you think that technology, like Anne4Care, improves health care?
- Would you recommend Anne4Care to others (i.e., peers)?
  - What are your reasons for recommending / not recommending?

*Specific questions for informal caregivers.*

- Do you expect a changing role as a caregiver due to Anne4Care?
  - What are the reasons to expect a change or not?
- Would you like to help in using and understanding Anne4Care?
  - How do you want to provide help?
  - Would you need help to assist in using Anne4Care?
    - If so, from whom would you like to receive help?

*Questions for the follow-up interview regarding technology.*

- What is your opinion on technology (in general)
  - What can technology bring for you? Do you imagine good things or also anxious things?
- What can technology mean for your health and health care?
- Which technologies do you currently use in your life and how often?
  - What is the reason for using these technologies?
- Which technologies would you like to use?
  - Is there anything in your life where technology could be helpful?
  - What should technology deliver?
  - What would you expect from the technology?

*Questions regarding reaching and (long-term) participation/collaboration with target group.*

- We would like to carry out this research in collaboration with you. Do you have ideas how we can do this?
- What was your reason to participate in this project?
  - How did you experience participation?
- How should we collaborate with you and other older adults with an immigration background during this or other projects?
- How could we reach more older adults with an immigration background?
- Through which communication channels could we reach you and other older adults with an immigration background?
  - Which messages should we share?
