# Supplementary material for: Acceptance of a Digital Assistant (Anne4Care) for Older Adult Immigrants Living With Dementia: Qualitative Descriptive Study
Source: JMIR Aging. 2024 Apr 19;7:e50219. doi: 10.2196/50219 (PMC11069095; doi:10.2196/50219)
Supplement: Multimedia Appendix 2 [file aging_v7i1e50219_app2.docx]

**Appendix 2. Interview guide – health care professionals of older adults with an immigration background**

This is the English translation of the interview guide that was used during the interviews with health care professionals. Some questions were also used during the interviews with the older adults if a health care professional was present and the question fitted the conversation.

*Introduction*

- Can you tell us something about yourself?
  - Age
  - Educational level
  - Nationality
  - Function
  - Work experience
- How does an average day look like for you in terms of providing care?
  - How often do you have contact with clients and informal care givers?
  - To what extent do you provide remote/care at a distance?
- What is important according to you when it comes to the health and well-being of clients?

*Use of (welfare) technology/digital tools in clients’ daily life and personal environment*

- Which technology/digital tools do clients use at your care organization or at home?
  - For example, mobile phone, fall prevention, alarm bell, video bell application

*Questions about Anne4Care.*

- Can you explain how Anne4Care has been delivered to clients so far?
  - What is striking about this?
  - What could be done better/different?
- How do you experience Anne4Care as a professional?
  - What advantages do you experience with Anne4Care?
  - What disadvantages do you experience with Anne4Care?
- Can you give illustrative examples of how you use Anne4Care?
- How would you improve Anne4Care?
- To what extent do you need Anne4Care? What is the added value for your clients and for you as a professional
  - For example day structure, maintaining social contacts, performing meaningful activities
- Do you need help with use or understanding of Anne4Care?
  - If so, with which aspects do you need help? Do you know by whom to receive help if needed?
- How do you expect the health situation of clients to change as a result of using Anne4Care?
- How do you expect that the care provided to clients will change through the use of Anne4Care?
- Would you recommend Anne4Care to other professionals?

*Questions regarding client–professional interaction*

- How do you prefer to build a relationship with your clients? How do you see the interaction and contact with your clients?
- How do you experience your relationship/interaction/contact with clients and informal care givers who use Anne4Care?
- Has the care you provided changed by using Anne4Care?
  - If so, what kind of change do you experience?
- Has the contact with clients/informal caregivers also changed?
  - If so, what kind of change do you experience?

*Questions regarding technology.*

- What technologies do you currently use and how often as part of care practices?
  - Which technologies would you like to use as part of care practices?
- What do you want to achieve by using technology as part of care practices?
- Do you expect technologies to change your relationship/contact/interaction with clients?
  - If so, how would it change?

*Questions regarding reaching and (long-term) participation/collaboration with target group.*

- How can we reach older adults with an immigration background to collaborate in research?
  - Through which communication channels and with which message?
- Do older adults with an immigration background have contact with others who have an immigration background?
  - If so, in what way and how often?
  - If not, do they want to have more connections with older adults with an immigration background?
  - How can older adults with an immigration background be involved in reaching the others?
- With which other professionals could we collaborate in this research?
