# Supplementary material for: Acceptance of a Digital Assistant (Anne4Care) for Older Adult Immigrants Living With Dementia: Qualitative Descriptive Study
Source: JMIR Aging. 2024 Apr 19;7:e50219. doi: 10.2196/50219 (PMC11069095; doi:10.2196/50219)
Supplement: Multimedia Appendix 3 [file aging_v7i1e50219_app3.docx]

**Appendix 3. Coding tree**

**
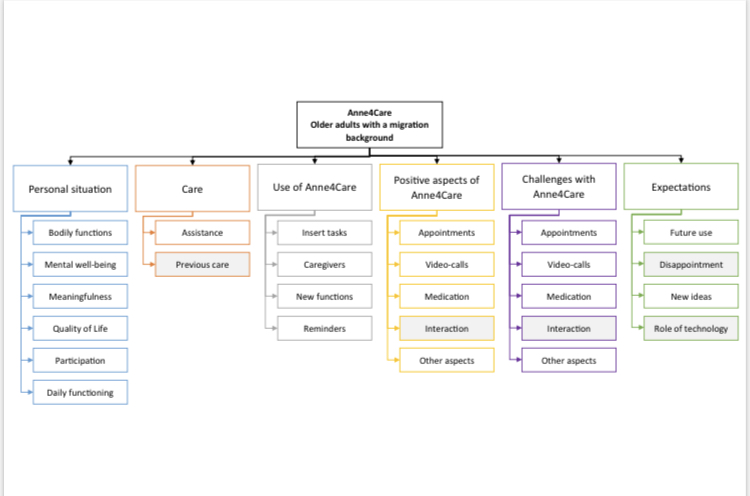
**

** Due to the conversations and active collaboration with the participant, the sub-themes previous care, interaction, disappointment, and role of technology were added to the coding.*
