# Supplementary figures and images for: Acceptance of a Digital Assistant (Anne4Care) for Older Adult Immigrants Living With Dementia: Qualitative Descriptive Study
Source: JMIR Aging. 2024 Apr 19;7:e50219. doi: 10.2196/50219 (PMC11069095; doi:10.2196/50219)

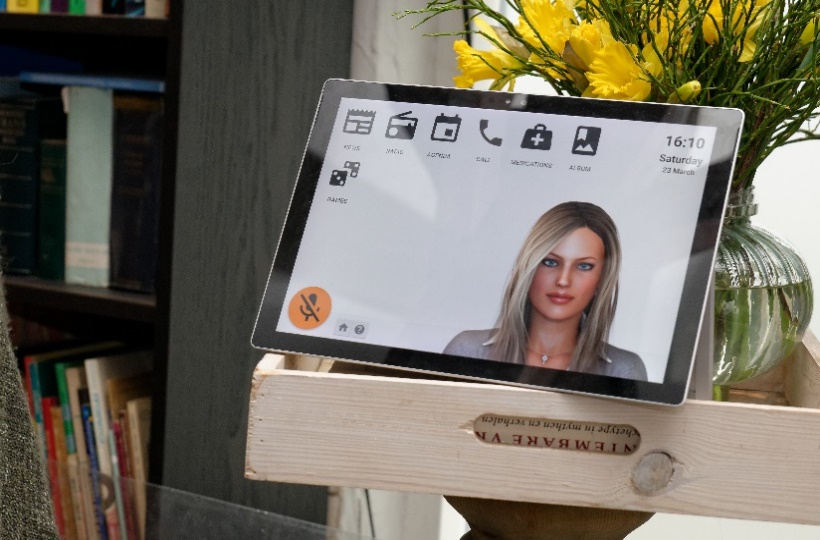
**
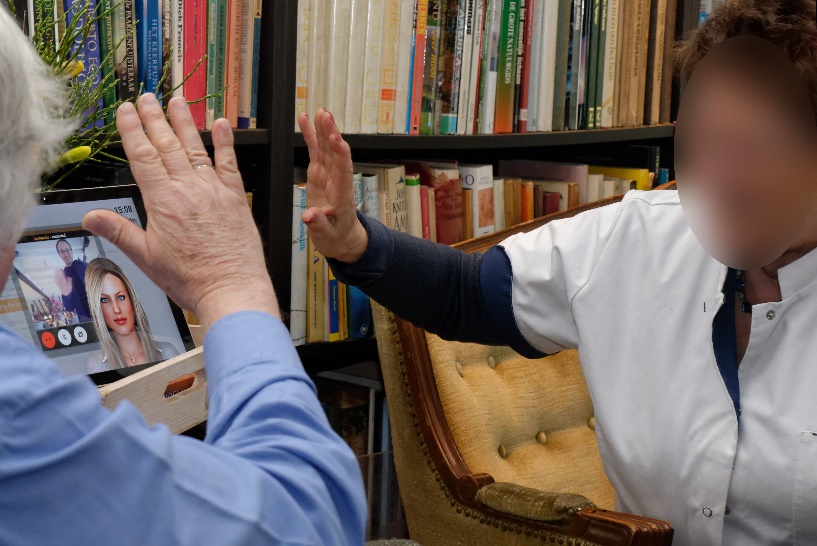
Multimedia Appendix 4. Visual information about Anne4Care**


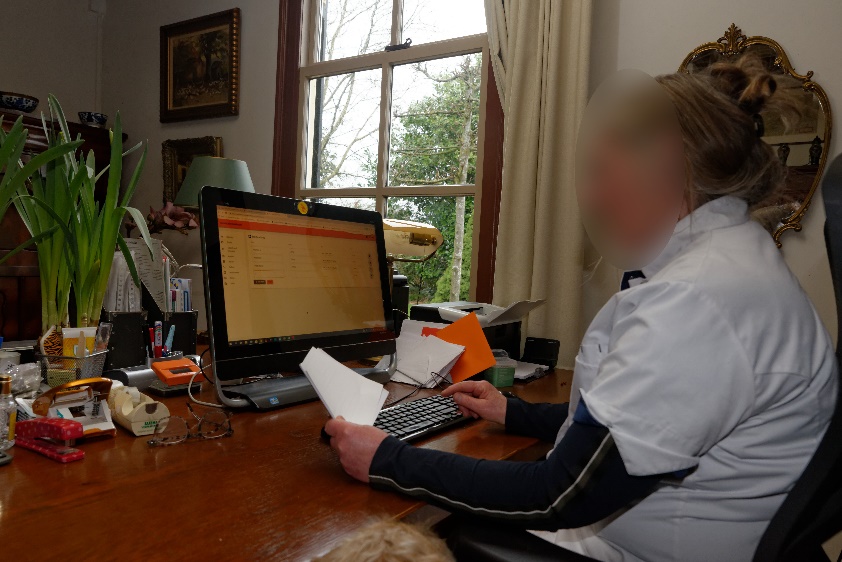

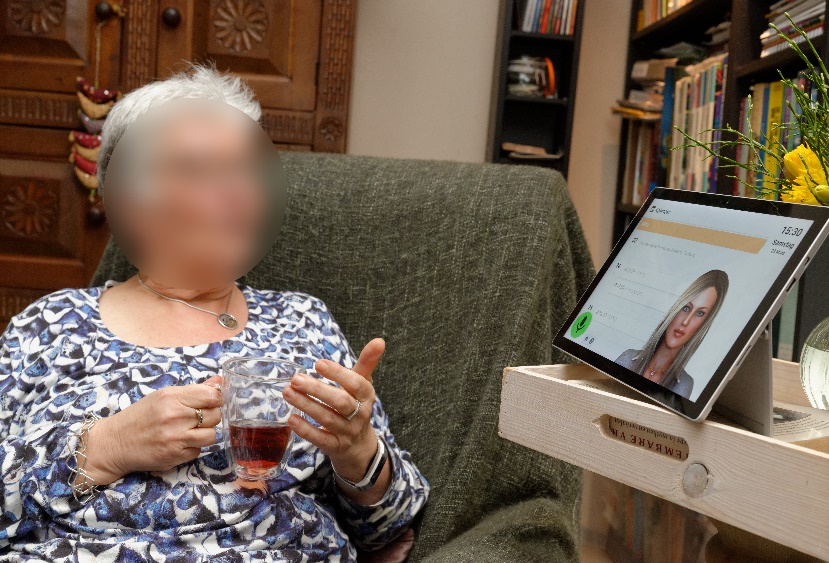

Supplement: Multimedia Appendix 4 [file aging_v7i1e50219_app4.docx]
